# Playback theatre in adult day centers: A creative group intervention for community-dwelling older adults

**DOI:** 10.1371/journal.pone.0239812

**Published:** 2020-10-01

**Authors:** Shoshi Keisari, Anat Gesser-Edelsburg, Dani Yaniv, Yuval Palgi

**Affiliations:** 1 Department of Gerontology, University of Haifa, Haifa, Israel; 2 School of Public Health and The Health and Risk Communication Research Centre, University of Haifa, Haifa, Israel; 3 School of Creative Arts Therapies, The Emili Sagol Creative Arts Therapies Research Centre, University of Haifa, Haifa, Israel; 4 The Centre for Research and Study of Aging, University of Haifa, Haifa, Israel; Institute of Mental Health, SINGAPORE

## Abstract

The literature indicates that theatre and drama-based interventions have the potential to improve older adults’ well-being and health. The goal of the current study was to characterize the process of a creative group intervention in adult day centers (ADCs), which integrates playback theatre and life-review principles. Our objective was to provide an evidence-informed framework for drama therapy interventions, which would allow older adults to bring up and explore their life-stories in a dramatic creative process in their own community. A total of 27 participants ranging in age from 63 to 91, took part in one of three playback theatre groups. The playback theatre group intervention comprised 12 weekly sessions. All sessions were videotaped to capture the lived experience of the creative process and were analyzed in post-intervention interviews. In addition, focus group meetings were conducted with 13 ADC staff members to decipher further the effects of the participation as perceived by people outside the groups. Three types of potential transformation were identified in the qualitative analysis: the evolution of life stories, evolution of playfulness, and expansion of social engagement. The results indicate the potential of the integrative framework to serve as a creative intervention in ADC communities, as well as its potential to bring about a personal transformation and expand it to enable a person’s social engagement in the community. The findings imply the potential benefits of using playback theatre groups to supplement the routine care provided in ADCs.

## Introduction

In recent years, efforts have been made to increase the care options of older adults in their own community. Adult day centers (ADCs) have emerged as a care option that allows individuals with functional limitations to receive care in the community while living at home [1]. ADC program models usually focus on the social and medical needs of the individual and family caregivers [2]. However, studies have indicated a specific fundamental need to develop interventions that directly focus on the enhancement of older adults’ mental health [3, 4]. According to the World Health Organization, *mental health* is "a state of well-being in which an individual realizes his or her own abilities, can cope with the normal stresses of life, can work productively and is able to make a contribution to his or her community" [5]. In this sense, the enhancement of mental health refers not only to the absence of mental illness, such as depression [6, 7], but primarily to an increase in levels of emotional, psychological, and social well-being [8, 9]. These interventions should be developed as programs that can be integrated into an ADC’s daily routine such that they will be accessible and encourage participation [10].

Accordingly, creative arts therapies may be a useful form of intervention that could be integrated into the daily routine of the ADC. These therapies employ an intentional and systematic utilization of arts to foster psychological growth. A growing body of research has established the positive impact of creative arts therapies in terms of improving various aspects of older adults’ mental health [11–16]. Visual arts, music, dance-movement, drama and theater provide additional means of self-exploration and expression within a therapeutic relationship [17]. In addition, engagement with the arts has been identified as a promising means of improving older adults’ well-being and health [18–20]. The creative process positions ADC members as active and productive contributors in their own communities, instead of as “patients” or “clients” [21]. However, the literature indicates that an essential need to develop evidence-informed models of effective arts therapies interventions for older adults still exists [13]. In particular, there is a dearth of research dealing with drama therapy and theater-based interventions with older adults [13, 22]. The current study explores the effect of group interventions conducted in ADCs, in which the life stories of older adults are recreated through their engagement in theatrical creative processes. The intervention focuses on Playback Theatre, a particular kind of non-scripted theater that combines artistic expression with an exploration of personal stories in a group dynamic process.

### Participation in theatre and drama in late life

The literature discusses the healing potential of older adults’ involvement and participation in theatre and drama [23, 24]. A pilot randomized control trial indicated that short programs, which include dramatic exercises, rehearsing, and performing, lead to the enhanced self-esteem, confidence, and happiness among older adults [25]. In addition, participation in theatrically-based interventions was found to increase cognitive functioning, such as memory, comprehension, as well as problem-solving ability [26, 27]. Furthermore, the few research reports on theatrical improvisation with older adults in dementia care found that such participation increased positive affect, reduced levels of depressive symptoms [28], and promoted learning, sociability, communication, and self-esteem [29].

An additional central aspect of the improvised dramatic engagement is related to the concept termed “playfulness” and “play” as a means of exploring the potential for expression, meaning-making, and relationship-building [30–32]. Playing is a central human experience that gives meaning to life [30], and unscripted imaginary play is a basic component of creativity, in which the authentic self is enabled to emerge [32]. The scarce literature on playfulness in old age indicates that it is associated with healthy aging and well-being [31, 33–35].

A recent review of the literature on older adults’ participation in theatre and drama illustrates its values in three areas [24, 36]: improved mental health and well-being; the provision of opportunities for learning and creative expression; and enhanced group relationships. However, this literature review indicates that an essential need exists to expand the academic research in the field in order to explore the manner in which participation in drama and theater in late life encourages transformation. The current study was concentrated on Playback Theatre, as a drama therapy approach that involves the intentional and systematic use of the theatrical/dramatic processes to achieve psychological growth and change [22].

### Playback theatre as drama therapy group

Playback theatre is a form of improvisational theater created in response to a personal story provided by the audience [37, 38]. A teller tells a personal story, such as a memory from childhood, a dream, or a personal experience. After the story has been told, a group of actors immediately improvise a theater piece, which reflects the story’s main themes [39]. Playback theatre was founded as a non-scripted, personal theatre with the intention of reviving the earlier goal of theater: to preserve memories and hold the tribe together [40]. As such, the theoretical background of playback theatre emphasizes the potential of a theatrical process to gather the community together through personal stories [41, 42]. Playback theatre takes place in performance settings, with trained actors enacting the stories of audience members, or in a group processes, where the group members become the actors of each other’s stories [38].

The improvised acting in playback theatre is based on empathy and spontaneous exploration as basic components of the process [38, 43, 44]. To understand and validate the teller’s point of view, the actors are required to use and develop their ability to imagine and identify with others’ subjective experiences [45–48]. In addition, spontaneity is considered to be an anchor in playback theatre, as a process that brings the individuals involved closer to their emotions, thoughts, and imagination [44, 49, 50]. Studies have indicated the value of playback theatre in promoting the well-being of the participants by enhancing self-esteem, self-knowledge, fun, and relaxation, and also as generating a sense of connection with others [47]. For example, older adults who practiced playback theatre experienced a significant improvement in their emotional well‐being after training [51].

Indeed, over the years, playback theatre has become a drama therapy group approach that is frequently used with varied populations, such as patients in hospitals and mental institutions [47], at-risk children [52], and older adults [16, 53]. Like other drama therapy approaches, having an intentional goal of fostering personal growth and transformation, the groups are facilitated by drama therapists in therapeutic settings [22]. Playback theatre groups has a ritual framework that defines the roles of the participants and the sequence of events [54]. In each session, one of the participants is invited by the conductor to share a personal story, which becomes the focus of the group meeting. Some of the other group members, as participant-actors, create a theatrical improvisation, which reflects some of the story’s content, without scripts or rehearsals. Thus, the teller becomes an observer and a witness of a spontaneous-creative reflection of his/her life-story [55]. In this way, playback theatre can provide a safe and creative space in which older adults can explore past experiences through their involvement in improvised theatrical action [39, 53]. On the basis of all these ideas, we developed an original creative group intervention in which playback theatre is integrated with the principles of *life-review* in old age [56–59].

### Implementing life-review in playback theatre groups for older adults

A vast literature points to the central role of processing one’s life story as vital to human development, especially in old age [60, 61]. Life-review is a common therapeutic approach for exploring and reconstructing the life story in late life. It leads to the creation of an integrative view of one’s life story by integrating past memories with present and future potential events and by including positive memories and achievements alongside the acceptance of failures and harsh life events [57, 59, 62]. In this way, life-review enhances the individual’s sense of integrity and acceptance of life [60, 61, 63]. Interventions that focus on life-review were found to effect older adults’ psychological well-being and mental health [58, 64–66]. Playback theatre as a process that focuses on the exploration of life stories can provide a creative atmosphere in which to implement the life-review principles through a “dramatic reality” [67].

The playback theatre groups examined here focused on working with significant life events as a means of succinctly crafting the life story in a time limited group process. The technique that is used is called *life-crossroads*, a specific life-review technique that uses three to five selected autobiographical memories, self-defining life events, or life periods in which an influential decision and/or a change occurred that had a meaningful effect on the individual's life course [14]. *Life-crossroads* emphasizes the idea that a life story is constructed by integrating influential decisions and meaningful changes that delineate one's life narrative.

In each group session, one of the participants shares one or several life-crossroads stories with the group. A few other group members, as participant-actors, with the therapist’s support and direction, create a dramatic improvisation, which reflects and reconstructs the life-crossroads’ themes. In one theatrical improvisation, the dramatic elements that occurred along an individual’s lifespan are gathered. In that sense, the improvisation includes theatrical representations of the past, present, and future, theatrical representations of harsh life events together with theatrical representations of coping resources and achievements.

Other studies that examined the integration of life-review using the concepts of life-crossroads with drama therapy [14] and playback theatre [16] indicated its value in terms of promoting meaning in life, self-acceptance, self-esteem, a sense of successful aging, positive relationships with others, and reduced depressive symptoms. These findings point to the healing potential of the integration of drama therapy, playback theatre and life-review. Our aims in the current study were to identify and characterize the process of playback theatre groups integrated with life-review, as an intervention in ADCs. Our objective was to provide an evidence-informed framework for drama therapy interventions, which would allow older adults to bring up and explore their life-stories in a dramatic creative process in their own community.

## Materials and method

### Research design

This qualitative study was based on a phenomenological perspective [68]. As such, it explored participants’ experiences from their own point of view. The study design was based on *arts-based research methodology*, which examines participants’ experiences through an understanding of their relationship with the artistic expression (in this case, dramatic improvisation) and its creative processes [69, 70]. In this study, dramatic expression was explored through the ways it embodies the life stories of older adults and reveals insights [71, 72] regarding past experiences, as well as present and potential future events. To capture the lived experience and its potential transformative effects [70], all the sessions were videotaped and analyzed with the participants.

#### Participants

The ADC settings recruited for this study were located in urban neighborhoods in Israel. These centers provide health and social services to community-dwelling older adults who are entitled to long-term care benefits provided by the National Insurance Institute because of their impaired psychological, physiological, and/or cognitive functioning. A homogenous sample [73, 74] of 27 participants aged between 63 and 91 years took part in one of three playback theatre groups, (10, 11, and 6 participants, respectively), each of which was conducted in a different ADC setting. The participants’ average age of 79.34 years; 19 of the participants were women, 14 were married, and 13 considered themselves religious. All the participants lived in urban neighborhoods. The participants fulfilled two inclusion criteria (see also [16]): they achieved a score of 25 or more on the Mini Mental State Examination (MMSE>24), indicating no cognitive challenges that could prevent participation [75] (the MMSE score was retrieved by the social worker of the center from records at the center), and they expressed an interest in participating in a theatre group and sharing their life stories with others. Post-intervention interviews were conducted with the groups participants who participated in at least 8 of the intervention’s 12 sessions. Two participants did not attend the interviews for medical reasons. Most of the participants had no previous experience of taking part in theater-like groups. In addition to the group participants, 13 ADC staff members of the 3 ADCs (6, 4, and 3 staff members, respectively) were interviewed in three post-intervention focus groups in order to validate the out-of-group effects of the intervention. Each focus group lasted 45 to 60 minutes and included the social worker who co-conducted the sessions and other staff members: the managers of each setting, the housemothers, and the occupational and activity instructors. Staff were recruited to the study by the manger, based on their involvement in the ADC community and their ability to evaluate the impact of the intervention outside the group in the day-to-day ADC life routine. For more information see Table 1.

**Table 1:** Participants.

Variable	Adult day center 1	Adult day center 2	Adult day center 3
**Number of participants in the group**	11 (one participant did not attend the interviews for medical reason)	11	7 (one participant did not attend the interviews for medical reasons
**Mean Age (range)**	78.27 (range 63–91)	79.36 (range 66–90)	81.00 (range 71–89)
**Gender**	63% female	73% female	91% female
**Place of birth**	70% in Asia and Africa; 10% in Europe; 10% in America; 10% in Israel	90.1% in Asia and Africa; 0.9% in Europe	16.6% in Asia and Africa; 16.6% in Europe; 66.8% in Israel
**Marital status**	36% living with a partner	73% living with a partner	33.33% living with a partner
**Education**	60% with elementary education; 30% with high-school education; 10% with tertiary education	73% with elementary education; 27% with high-school education	33.33% with elementary education; 66.66% with high-school education
**Religious**	54% consider themselves orthodox	64% consider themselves orthodox	16.66% consider themselves orthodox
**Previous experience with theatre**	None	One participant took part in a theatre group	One participant took part in a testimony theatre activity

#### Procedure

An individual introductory meeting was conducted with each of the participants before the intervention began in order to introduce them to the research procedure and receive their written consent. The playback theatre intervention consisted of 12 one-and-a-half-hour weekly group sessions, co-conducted by a drama therapist and the ADC’s social worker. Each session consisted of four parts. Table 2 details each part of the session.

**Table 2:** Session structure.

Stage	Description
Warm-up session	• Varied dramatic and movement-related experiences to warm up the participants’ spontaneity and help them enter the realm of dramatic reality • The various exercises include improvisational theatrical exercises, a mirror game [76], crafts, an imaginary telephone conversation, songs that accompanied their life, etc.
Sharing life-crossroads stories	One of the participants responds to the therapist’s invitation and becomes “the teller,” sharing one or several of his/her life-crossroads story, which are integrated into one life story
The theatrical improvisation	• Some group participants, with the help of the conductor, co-create an improvisation that reflects some of the story's content • The conductors helped to evolve the participants’ improvised acting using dramatic exercises, doubling [77], and the “playback theatre short forms” that transform the personal stories into an aesthetic theatre piece [38, 39]. • The teller witnesses and observes the improvisation from the point of view of a “spectator” • According to the principles of life-review, the theatrical improvisation created by the group usually simultaneously represents different phases of the life course: dramatic roles from childhood together with dramatic roles from adulthood and old age • The theatrical improvisation also strives to combine dramatic roles that represent achievements, positive memories, and coping resources together with dramatic roles that represent disappointments and harsh life events • At the end of the performance, guided by the conductor, the teller responds to the theatrical improvisation • The teller can also re-direct the improvisation and the way it reconstructs his/her story
The sharing circle	• The group participants are invited to respond to the story and the theatrical improvisation from a subjective point of view, i.e., sharing their own emotions, associations, etc.

All sessions were video recorded using two cameras: one camera recorded the teller, and the second camera recorded the improvisation that was created in response to the teller’s story. 36 sessions of three group processes were filmed, each using two cameras, producing a total of 72 videos per group. The length of each video was approximately 100 to 115 minutes. After each session, the conductors wrote notes, that described the main content and significant moments that emerged in each session, as well as their feelings and thoughts about the process. After the final meeting, the researchers watched the videos and read the conductors’ notes. The notes helped identify the meaningful parts of the videos to be analyzed with the participants during the interviews. For each participant, they selected the parts of the video that showed their story’s improvisation and the parts where they improvised in response to others’ life story, as well as other significant parts related to them. Two weeks after the group’s final meeting, the therapist from the research team met each of the participants in the ADC in a semi-structured interview, during which they watched the videos together. The duration of the semi-structured interviews was from 45 to 105 minutes. Watching the videos together captured the lived experience of the creative process in all its intimacy, depth, and reflective atmosphere. This assisted participants to reflect on their experience. The videos were analyzed with the participants, embracing an attitude that made the participants researchers of their own process and the way it affected them [70, 78]. Based on a comprehensive process analysis [79], the interview focused on the significant moments of the process that ultimately led to a transformation in the participant's subjective experience. In addition, after the group’s final meeting, a focus group meeting was conducted with the ADC staff members to decipher the effects of the intervention outside the group in the ADC. This research study was approved by the Ethics Committee of the Faculty of Social Welfare and Health Sciences at the University of Haifa, Israel (Confirmation number 197/17). To preserve confidentiality, pseudonyms are used for the participants and staff members.

### Data analysis

The data included in the analyses were the participants interviews and the focus group meetings with the staff members. As mentioned, the video recordings and the conductors’ notes helped to capture the lived experience of the creative process during the interviews. The data were analyzed using a thematic analysis method [80]. To ensure reliability, two members of the research team—S.K and A.G (first and second authors), conducted an independent analysis of the data, each of whom began the analysis with a read-through of the transcripts to understand the general and potential meanings. An initial coding structure was created, based on the descriptive coding that resulted from coding units of text as themes. Examples of coding units were “sharing the life-story,” “the embodied story,” “acknowledgment,” “self-expression,” “shared experience,” and “social engagement.” The researchers worked systematically through the entire data set, giving full and equal attention to each data item, and identified interesting aspects in the data items that might form the basis for themes across the data. The following phase involved the organization and collation of all the potentially relevant coded data extracts into themes [81]. In the subsequent stage, the researchers met and, through an iterative process of reading and rereading the text, subjected the codes to constant comparative analysis. The themes were analyzed in relation to the coded extracts and the entire data set to generate a thematic map of the analysis. These stages included further refinement of the interpretation, definition, and naming of the themes, the coding structure, and the patterns and relationships across themes [80, 82]. The result was a detailed coding structure for categories and sub-categories, agreed upon by both researchers and a third researcher, the last author, to evaluate the thematic map. In the following stage, the structure was introduced and considered by the third author, to evaluate and refine the analysis further. Finally, the themes were presented to the participants and staff members who then evaluated the findings. We worked in units of pairs and trios, and the thematic map was introduced to each unit. Due to participants’ vision and reading difficulties, we adjusted the evaluation feedback using an enlarged chart and reading the themes and quotes to the participants. Afterwards, the researchers and participants discussed the results together.

## Results

**Fig 1:**
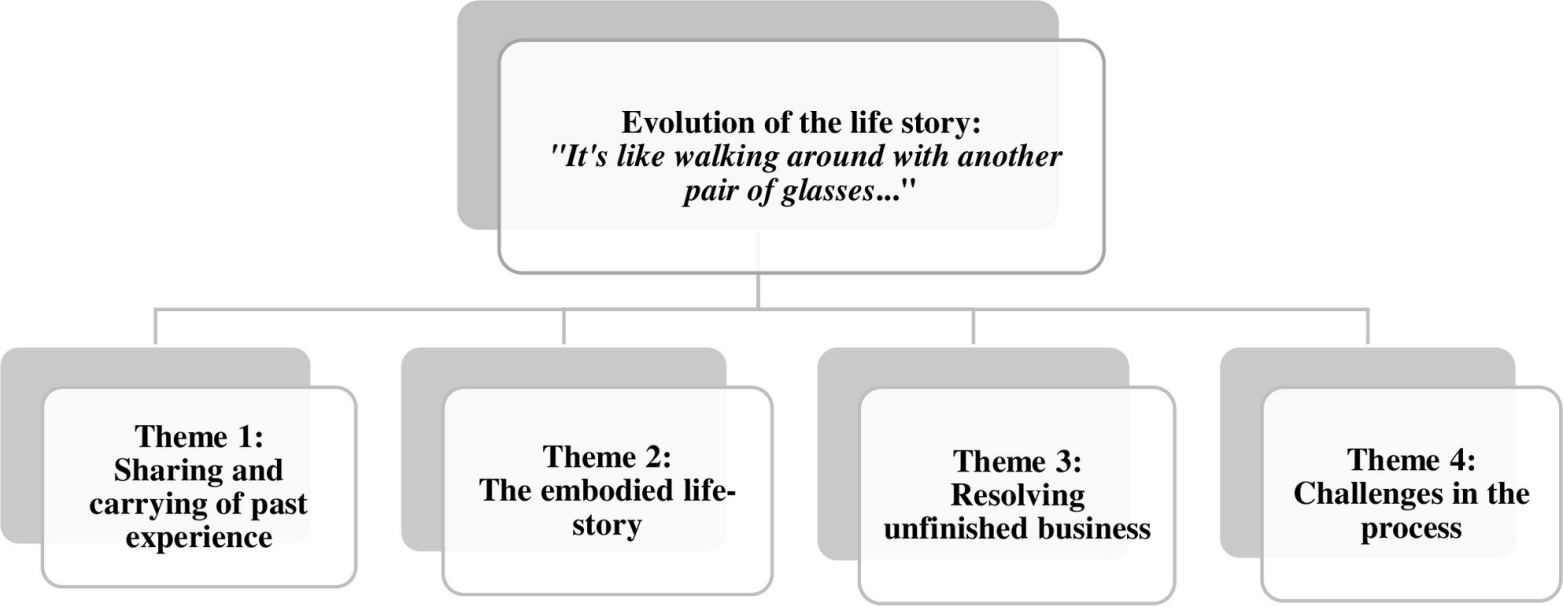
Evolution of the life story.

**Fig 2:**
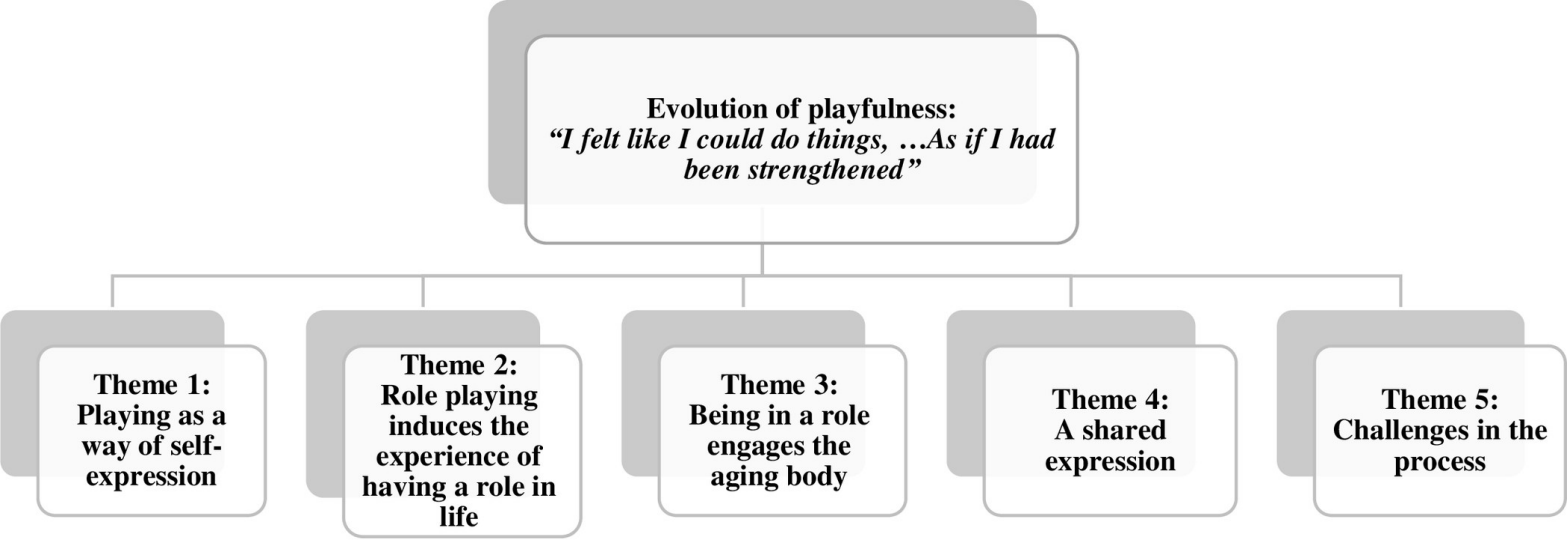
Evolution of playfulness.

**Fig 3:**
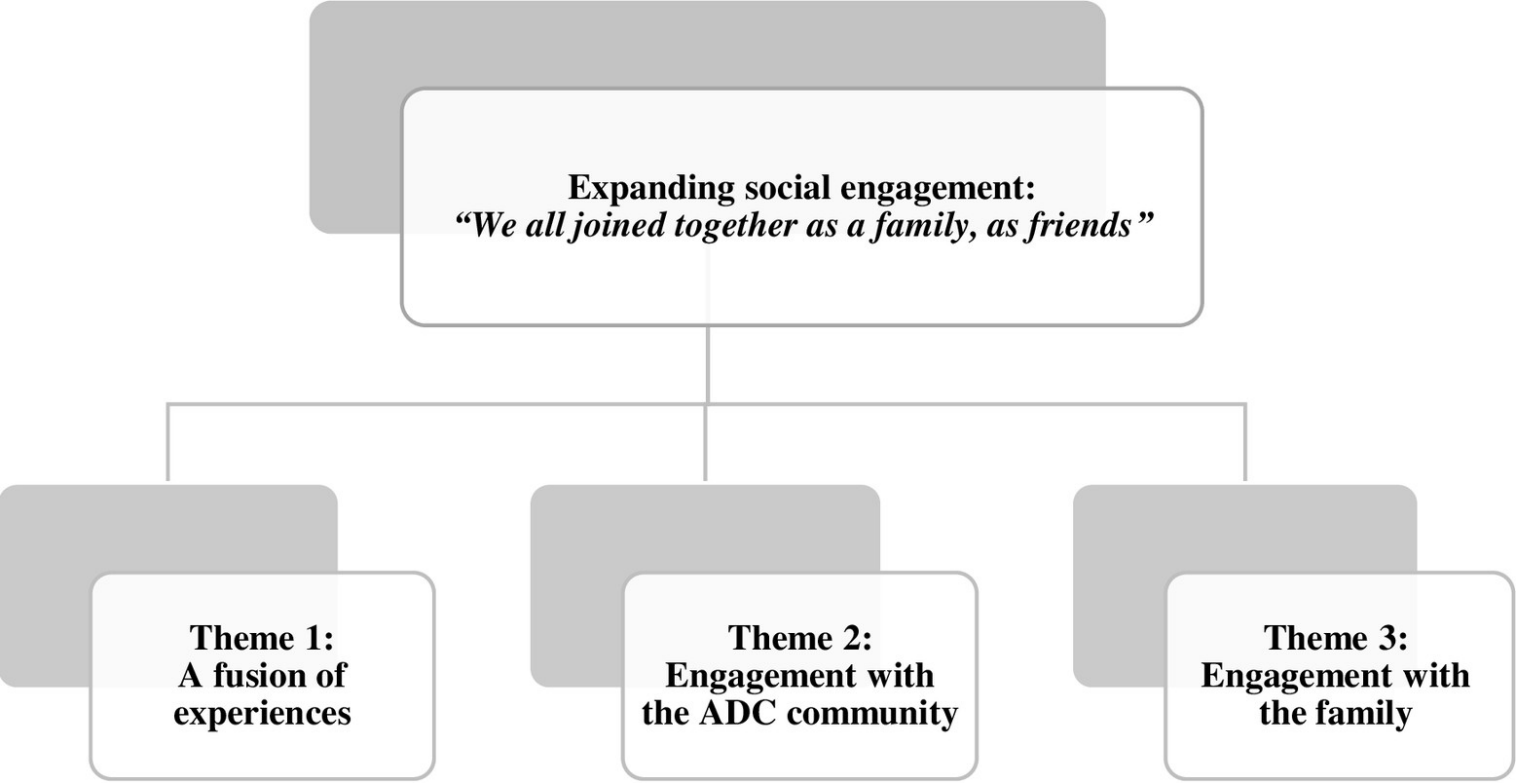
Expansion of social engagement.

Three main categories emerged from the experiences reported in the data (Figs 1–3). The categories contain the potential transformations that occurred during the process: (a) evolution of the life story; (b) evolution of playfulness; (c) expansion of social engagement.

### The evolution of the life story: “It's like walking around with another pair of glasses…”

This category describes the evolution of a life-story along the process and it reveals four themes: 1) Sharing and carrying of past experiences, 2) The embodied life-story, 3) Resolving unfinished business, and 4) Challenges in the process. Additional supportive data for this category are presented in Table 3.

**Table 3:** Supportive data for category 1: The evolution of the life-story.

Theme	Quotes
**Theme 1: Sharing and carrying of past experiences**	*It’s that it [the acting] kind of recreates the past*. *Yes*. *It tells the stories*. *Because if it was not there (the acting)*, *people might share their stories less with the group*, *and they would keep things to themselves… Sometimes*, *people take their stories with them to the next world*, *but here they gave some of the stories a chance to be told and they were able to let them out*. (Benny, an 82-year-old man)
	*What I think*.* *.* *. *is that*.* *.* *. *there is drama and there is acting and all that*, *and maybe the acting and making [the story] into a play*.* *.* *. *this way they will connect to it*, *from the theater*. *And it enables them to talk about things*, *to tell things*.* *.* *. (Daniella the housemother)
	*So*, *I shared my story*. *It was such therapy*, *to let it all out and feel good about telling it*. *And it really did me good to share and to present myself*. *Usually I don't share things with others*, *but here I did*. (Tina, a 76-year-old woman)
	*There were sad stories*, *there were happy stories too*.* *.* *. *I never thought to mention the story of my brother*.* *.* *. *But as soon as Sarah played the song about the “soldiers” I lit up and the story evoked… That's it*, *and it did me good that I shared …*. *It was very good*.* *.* *. *They got to know the story about a child that who was born in a tent*, *without any help*, *not even a midwife*, *without a hospital*, *and came to be what he became*.* *.* *. *Now they know*. (Hanna, an 83-year-old woman)
**Theme 2: The embodied life story**	*It evokes the things that were in the past*, *just evokes the whole picture*. *It brings me back*.* *.* *. *to the pink dress*, *to my cropped hair… You see this whole picture in front of your eyes*. *Chocolate on the table in the shack*, *or the presents he used to bring me*.* *.* *. (Ronna, an 81-year-old woman)
	*It's like a replay*, *a movie*.* *.* *. *and I couldn't believe it was all*.* *.* *. *A play that is real*, *it really is*, *and everyone understands each other more*. (Hedva, a 65-year-old woman)
	*The acting*. *It's something that takes them to the depths of things that can't be reached in any other way*.* *.* *. *it gives some perspective to things*.* *.* *. (Nurit, the social worker)
	*It came back to me*, *the story I had*. *Suddenly like something*.* *.* *. *I think something real is coming back*, *imagining like*.* *.* *. *the people*, *what they said and how they acted…that's how it was*, *so I smiled*, *I was happy with it*.* *.* *. *It gave me something*, *you know*, *so I smiled and rejoiced … They were playing just like we were*, *it is a kind of inspiration [a path to the past]*.* *.* *.* *. *to what used to be*. (Tina, a 76-year-old woman)
**Theme 3: Resolving unfinished business**	Mary, an 82-year-old woman thanked and asked for a forgiveness from a man she was in touch with when she was young, years ago in her homeland:
	*I can come in nicely*.* *.* *. *thank him for what he did for us*.* *.* *. *and he will feel good*, *and I will feel*.* *.* *. *he will not feel angry at us*, *that he will not hate us*. *Do you understand*? *[I would say]*: *"I didn't set you up*, *I just wanted your favor*, *and I had no choice*.*" I didn't do anything wrong…I wanted to have a closure*.
	David, a 90-year-old man, explained how the process allowed him to recall and tell things to his young love, a young woman he was supposed to marry but was killed in the war before they could meet again:
	*Yes*, *that's right*, *I had to let go*. *Now my life goes on*. *They say*: *"Time heals all injuries*. *Time passed*. *After a few years*, *I got married*, *had children*, *grandchildren*, *great-grandchildren*, *and now everything is moving*. *Yet*, *while thinking about her*, *how I used to be*, *I loved her a lot*, *there was love*.* *.* *. *a very big love*, *and I lost her*.* *.* *. *but time passes*.* *.* *. *and you [I] wished to tell [her]—that I did things and I was able to start a family again only ten years later*.* *.* *. *This is my fate*. *Just like that*.
	Bina, an 80-year-old woman, wanted to study medicine and be a doctor, but in the end, she could not go to the university and she became a teacher. Her playback concentrated on the choice she had to make.
	*I really wanted to study*, *and I wanted a specific subject*, *medicine*. *It was very important to me*, *and even today I’m involved with it*, *I read medical brochures*, *I’m involved with all kinds of things*.* *.* *.* *. *The very thing I came up with is that I would not study medicine*. *But I would go for something quite similar*, *and it should satisfy me*, *and it provided me*.* *.* *. *And on the group stage [during the improvisation] I saw that yes*, *I accepted it*, *I realized that this was my decision and it was good*.
	Nurit, the social worker, responded to Bina’s life-story and the theatre improvisation that was created in response to it:
	*Sometimes*, *I thought I gained much more from the process than they did*.* *.* *.* *. *It gives some perspective to things*.* *.* *. *For example*, *Bina*. *She had a dream*.* *.* *. *she wanted to be a doctor*, *and in the end*, *she worked with children*, *and you can see [in the playback] how she helped the children*, *cared for them and that*.* *.* *. *But let's say I compare myself to her*. *I never wanted to be a social worker*, *I wanted to be an engineer*.* *.* *.*but at that point [in that session]*, *I realized something*, *and then I told myself that "no matter what a good engineer you could have been*, *you could never do what you do today [as a social worker]*.*" As if for me*, *it sorted out something in my head*.* *.* *.* *.
	Rebeca, an 82-year-old woman, shared how the playback theatre group helped her process her grief after her husband passed away. In this quote she explained how the dramatic reality allowed her to have a conversation with her deceased husband:
	*I felt like he was really alive*, *and I was talking to him; I felt better*, *like he was alive as always and I was talking to him*. *It was good*. *It was really good*, *I felt like I can relieve my heart a little bit*: *“Where are you*? *Why don't you come*? *Why did you leave me*? *You went too early; you didn't tell me you*.* *.* *.*” I didn’t make it; I didn’t know he was going to die…*
	When she watched the video where one of the participants was acting “Rebeca the child” who participated in the Jewish underground in one of the Arabic countries in the 50’s, she added:
	*When I see myself in the theatre [improvisation] it does me good*, *I can feel myself; this is me*. *I say*: *“Here I am*,*” and it does me good*. *I remembered [the child I was]–I was so diligent*, *I was in the underground…and I wasn’t scared…and it helped me…and I realized how I overcame all these difficulties… [today also] and told myself—I want to survive*, *to overcome my problems*, *to see the grandchildren*, *the children*, *I want to be at their weddings*. *Between me and myself*, *[I realized] I don’t want to die*, *to follow him*. *I want to live*.
**Theme 4: Challenges in the process**	Marina, a 66-year-old woman, explained that she would prefer a longer process, so she could share and process more stories with the group:
	*And I would love it*, *no matter when*, *if we could have that group again*. *Because I haven't told all my stories yet*, *I haven't told them yet*.
	*You gave us the opportunity to tell only one [story] each*, *you know*? *A story that we kept inside*. *Yet*, *I know very well that everyone has more stories and lots of stories that could be shared*.* *.* *. (Hanna, an 83-year-old woman)
	Enya, an 89-year-old woman, reflected on the challenges she experienced regarding acting in difficult stories:
	*If he (the actor) is a sensitive person and lives what his friend has been through*, *it's not easy*.

#### Theme 1: Sharing and carrying of past experiences

According to most of the participants, knowing that the theatrical dramatic creative process engages all the group members in their life story encouraged them to bring their personal stories to the group, which created a safe and caring atmosphere within which to treat the emerging life-crossroads stories.

“I think the fact that we played their [life-story] characters gave everyone the opportunity to open up…it affected everyone… It gave them support… encouragement” (Bina, an 80-year-old woman)

“The acting itself gave unity to the group, if you had just said: ‘I want to tell my story,’ [then] 50 percent of the talking would have ‘disappeared into thin air.’ But here… you [I] really share…then they [the group participants] create a theatre about the things I've been going through, and I feel they care. It made them participate with me… they made a special effort to show me myself… So, I could really tell [my story].” (Marina, a 66-years-old woman)

#### Theme 2: The embodied life story

Most of the participants described how the theatrical improvisation brought to life a past memory and transformed it into a vivid, living experience. The participants described how the tangible aspects of the dramatic play awakened the old story in the “here and now” of the group process and fused it with the new shared experience. Rebeca, an 82-year-old woman, explained:

“When they recreated my story, I went back to the past, and I felt like I was really there, watching what I went through…I could see it in reality, I could see it as a dream…. I saw everything again: how I moved, how it was, how it is… It made me feel good when I saw it, because finally I had other people there with me who knew my story, and I wasn’t alone, I'm not alone… I could feel myself; this is me… So, I say–here, this is me, and it does me good.”

The teller, as a spectator of her own life-story, watched the improvisation from the sidelines. This quote demonstrates how the improvisation simultaneously embraced both reality and dreams, memories together with present and future events. In addition, the teller explained how the theatrical improvisation created an experience of acknowledgment and validation of the life she had lived.

Victor, a 63-year-old man, explained how the dramatic improvisation that was created by the other group participants brought to life new perspectives for his life story in a manner that enhanced self-acceptance:

“When they presented my story, I could see it from the outside. It’s very realistic; you can see things differently. It's like walking around with another pair of glasses, seeing things from another angle, to reach different conclusions… watching their acting, I realized that, despite everything, I am still here.”

Through the vivid, live improvisation, a new experience was constructed, which also helped with resolving "unfinished business."

#### Theme 3: Resolving unfinished business

The participants brought to the group process life-crossroads stories that contain unfinished conflicts from the past. The theatrical creation’s flexibility allowed the participants to explore alternative versions of their life story in a way that helped reconstruct their inner experience. The process around Simon’s, a 78-year-old man, life-crossroads story illustrates this idea.

Simon shared his life-crossroads story of the loss of his younger brother. Simon’s family immigrated to Israel when he was 8 years old. One day, a few weeks after they had arrived at the refugee camp, Simon went out to play with his friends and failed to notice that his brother had also left the tent and followed him. Later, the brother was found dead in a hole. Simon described the guilt that accompanied him over the years as a result of not being able to save his brother. In response to his story, the group improvised three scenes: Simon’s family after immigration with their hopes for a new life, the grief following the loss of his brother, and the growth of the family years later. The ability of the group to take on the dramatic roles of Simon and his family members and to contain the difficult emotions through movements and text enabled Simon to re-explore his story for the first time in 70 years. During the improvisation, Simon first experienced the acceptance of the group participants, who, without blaming him, validated his guilt and emotional suffering.

“It was hard for me, but I had to tell this story because… it kept chasing me… and here [in the improvisation] no one said: ‘Why didn’t you stop him from running away?’… no one [from the group] yelled at me… that set me free… because my whole life it has haunted me, the case. [I would ask myself]: ‘Why did it have to happen and where did he go anyway?’ Now, I think I realize that we couldn’t have prevented it, there was nothing we could have done… I have no answer. It happened, and no one could stop it, and no one can bring him back to me.”

This quote demonstrates how the guilt was constructed through the social environment that surrounded Simon when he was a boy, and how the new experience of acceptance and liberation from his long-standing guilt was constructed through the recreation of the story in the group.

#### Theme 4: Challenges in the process

It is important to mention that the process of reliving self-defining memories in the group sometimes includes the sharing of traumatic life events. The participants in the group reflected on the difficult emotions they experienced regarding the traumatic stories they heard in the group:

“I heard stories of people. I didn't think, really. A story hurt me and a story… Almost everything hurts, it hurts. I didn't know there were such things, you know?. The girl. Her dad used to beat her up… This is trauma… I heard stories that… I had, I was amazed, I didn't know there was a world like this.” Mary (an 84-years-old woman)

This quote indicates the importance of allowing time and space in the sessions to process difficult emotions and thoughts that arise in response to the harsh stories and performances. Another challenging issue for the participants was the limited length of the process, with only 12 sessions, and most of the participants felt they would like to share more stories with the group:

“At that age…there are many experiences, everyone has many experiences, and we were only able to tell… very little of what we went through. For example, I have a lot of stories I can tell, but there was no time…” Benny (an 82-years-old man).

This demonstrates the need of the participants to share their stories with others. This quote also illustrates the significance of the group for the participants. The following category describes the dramatic improvisation and the evolving playfulness, as another meaningful transformative aspect in the process.

### Evolution of playfulness: “I felt like I could do things…as if I had been strengthened”

This category describes the evolution of playfulness along the process and it reveals five themes: 1) Playing as a means of self-expression, 2) Role playing as inducing the experience of having a role in life, 3) Being in a role as engaging the aging body, 4) A shared expression, and 5) Challenges in the process. Additional supportive data for this category are presented in Table 4.

**Table 4:** Supportive data for category 2: Evolution of playfulness.

Theme	Quotes
**Theme 1: Playing as a means of self-expression**	*It is the first time [that I acted]*.* *.* *. *I was afraid it might not work*, *maybe I wouldn't do it right*. *Then I slowly started*.* *.* *. *yes*. *For the first time*.* *.* *. *I'm doing the*.* *.* *. *acting*.* *.* *. *and dancing in a group*. (Rosa, a 76-year-old woman)
	*I didn't exactly have a text or something I could turn to*.* *.* *. *And I was very shy*, *but slowly I opened my mouth*, *and I spoke*, *and I presented and was not ashamed anymore*. (Reuven, an 80-year-old man)
	*I had the pleasure of attending and acting*. *And I enjoy it*, *I love it*. *I like to participate and be active*, *and I'm not ashamed and I*, *I did everything I wanted*. *I*, *if I could*, *I tried*. *And I had fun*… (Mona, an 83-year-old woman)
	*Look at Simon*, *for example*. *He became more open*.* *.* *. *You can see the change in his verbal ability*.* *.* *. *in his ability to contain others’ stories*, *to hold their sentences*.* *.* *.* *. *in his ability today to share*, *to come to the day center and look for a joke to tell*, *so others will laugh*. (Marta, the adult day center manager)
**Theme 2: Role playing as inducing the experience of having a role in life**	*I felt good that I participated too*.* *.* *. *I acted according to the person [to his story] together [with the group]*. *It feels*.* *.* *. *good*, *a good feeling and I felt like that in my heart*, *like I did something important too*.* *.* *. *I participated with everyone* (Nira, an 82-year-old woman)
	*I don't want to say it raises my blood pressure because I have high blood pressure (laughing)*.* *.* *. *but it raises my humanity*, *my heart beat rises*, *because I love it*, *love it*, *how they look at me [when I act]*, *and how I present myself and how I came to know the people when I was acting*. (Simon, a 78-year-old man)
	*So*, *we acted*, *played what was needed [according to the story]*. *At that moment*, *we also had the courage*, *because we were in the story*.* *.* *. *and the play was good*.* *.* *.* *. *Everyone thought not of herself*, *[but] of the character she was playing*, *she played it beautifully*. *They are not actors*, *are they*? *Very simple older adults who come to the day center*. *And they acted as much as possible*, *and it was successful*.* *.* *.* *. (Hanna, an 83-year-old woman)
	*I think for the first meeting or two that Yochi*.* *.* *. *he usually does not express himself outside*. *Normally*, *he does not participate in groups*.* *.* *. *he sits on the side*. *Then*, *suddenly he got up*, *he was the first to get up to act*.* *.* *. *He had to want something very much to get up like that and act*. *Because before that*, *we were always encouraging him [to participate]*.* *.* *. *and suddenly he volunteered*, *yes*, *on his own* (Nurit, the social worker)
**Theme 3: Being in a role as engaging the aging body**	*Something is amazing*.* *.* *. *Listen*, *we have now received an application from the National Insurance*.* *.* *. *They reported about one of the participants here*, *that he had a major reduction in functioning*.* *.* *. *But here in the group*, *they [the social worker and the participant himself] tell me that he is standing*, *acting*, *holding the stories*.* *.* *. (Marta, the adult day center manager)
	*I felt free*, *I felt that I had strength*, *strength*, *that I could say and act whatever is needed*.* *.* *. *it strengthened me*, *strengthened and more…* (Hava, a 78-year-old woman)
	*We would sing*, *we would dance*.* *.* *. *act for them*.* *.* *.* *. *It did me good*, *I remembered*, *I remembered everything like when I was young*. *It reminded me of myself as a young child*.* *.* *. *when I was dancing and doing things… like I used to do…*(Rosa, a 76-year-old woman)
	*I don't stand much*, *it's hard for me*, *I get tired*.* *.* *. *but in the play–Yes*. *[Because] if I sit down it's not exactly right*. *(Watching the video)*. *Here*, *I am like him in the mines [like the teller]*. *So*, *I stand*. *He [the boy in his story] stood*, *a child works in the mines*, *so did I [in the improvisation]*. (Dina, an 83-year-old woman)
**Theme 4: The shared expression**	Simon, a 78-year-old man, described the feeling of watching the participant-actors improvising in a response to his story, when he told the group about the loss of his younger brother:
	*I felt like they were participating with me*, *sharing the loss with me*, *and the sadness and the detachment…*
	*I see that they identify with me*, *they tell my story*, *it takes me back to that period*. *They did it very nicely*.* *.* *. *I see that they identify with me*, *that they understand me*. *It's beautiful*.* *.* *. *you have to get into it*, *to feel like you’re in the person's shoes*.* *.* *. *to be in his position*.* *.* *.* *. *It's like I lived it*, *got into their shoes*, *saw what they went through*.* *.* *.* *. *It's more real with the theatre*… *In this play*, *you really get into her situation*. (Lia, a 75-year-old woman)
	*What the [participant-actor] said was true*.* *.* *. *I felt he was saying exactly what I wanted to say*. *It's my inner voice*, *what I didn't say [in real life]*.* *.* *.* *. (Hedva, a 65-year-old woman)
	*It more gives unity and connectedness*.* *.* *. *They feel and play what I felt*, *and they tried to make me forget [the pain]*, *to make it easier for me*.* *.* *. *Yes*, *it makes it easier that they told the story [even] that they didn't see it*, *and they were not there*. *They acted precisely how it was as if they were there*.* *.* *. *as if they were*. *It means something*. (Zohara, a 77-year-old woman)
**Theme 5: Challenges in the process**	Hedva, a 65-year-old woman, explained that sometimes the acting was a bit lacking and deviated from the original story:
	.. *I sometimes say that maybe she was missing something*, *maybe if she had done it differently*, *maybe if she had taken these steps*.* *.* *. *I mean*, *maybe it was a little different*.* *.* *. *I think people have no experience in that [acting]*.* *.* *. *I’ll tell you what; it might be that if [they could] see the text [before]*, *read*, *understand it*, *then we would act it better*
	Victor, a 63-year-old man, explained that the improvisation’s divergence from the original story allowed him a new perspective:
	*Because everyone always sees things from their own aspect*, *from their own point of view*. *So*, *there may be some divergence [from the original story]*.* *.* *. *small*, *but to me*, *it can only give more perspectives for solving the problem or proceeding better*.

#### Theme 1: Playing as a means of self-expression

The interviewees reported a positive affect directly related to the dramatic acting itself as a form of self-expression that involves a sense of pleasure, release, creativity, and humor and, for most of them, it was a first-time experience, as shared by Mary (an 84-years-old woman):

“How I love, I love (to play). I felt good. And I had never acted before, maybe once…when I was in the fourth grade… I think we performed Cinderella.”

Play-acting also created a sense of capability, which influenced the participants’ sense of self-development and self-expression. As Dina (an 82-years-old woman) explained:

“I felt like I could do things … I started to talk more, to be more open. . . . I started to get stronger. So, instead of being ashamed, I began to let things out. As if I’d been strengthened, I began to let go of the things that I had always been ashamed of…”

This describes how the improvised acting enhanced the participant’s self-expression on stage and afterwards in the community. This idea was also reflected in the ADC community, as Marta, the ADC manager explained:

“There were people who came to me later and opened up, [telling me] much more than I had ever known about them…this is because they took part in those [playback theatre] sessions… They learn that they can share things, and there are people who will listen and really be there for them.”

The experience of taking on a dramatic role in the theatrical improvisation also induced the experience of having a role in life.

#### Theme 2: Role playing as inducing the experience of having a role in life

The participants indicated that one of the main aspects of their positive experience was based on the ability to take on a role, to become part of a group theatrical creation; that is, the ability to be “on call” and to succeed in it is a basic situation that reflects being engaged with life. As David, a 90-year-old man, described:

“I pretend I am living in this story; I act as if it were really happening now…I feel proud, yes, yes…[that] I managed to play my part properly… I feel like I am someone.”

Rebeca (an 82-year-old woman) explained that playing in the life-stories of others brought to life her own past experiences, when she had a meaningful role in life, helping others as her siblings’ caregiver.

“It’s like…moving backward and forward in a movie. Here, I feel like I am young again, and I help others…I feel like I am [once again] doing it for my brothers and sisters…taking care of them, like I used to…70 years ago.”

The experience of feeling relevant and needed enhanced the participants’ commitment to the process, expressed by the participants’ consistent presence in the sessions. For instance, in the 12 sessions that comprised the process, the average participation rate for each participant was 10 or more sessions (for the 3 groups, the average for participation was 10.45, 11.18, and 11.28 sessions-per-participant, respectively). As Nora, an ADC manager, explained:

“It’s not natural for us to have a consistent presence… This is an indicator, because our members find it difficult to participate [in groups] on a permanent basis… They have a doctor's appointment, they need to buy things, take care of their grandchildren… They probably made a conscious effort to arrive consistently. We felt they wanted to come—for this group… This morning, Sara (one of the participants) told me: ‘I had to come today.’”

#### Theme 3: Being in a role engages the aging body

The participants related to the dramatic playing as a creative process that engaged the aging body, which was required to be part of the theatrical creation, as Zohara, a 77-year-old woman, explained:

“This [acting] makes me feel the way I used to feel… It [the experience] reminds me of a time when I was full of energy, my legs, my hands, everything functioned… I've become totally different from what I once was… (on stage) They could see me more, see me more freely… I could stand, and I could walk with the walker, that makes me feel good, that I just could be.”

The aging body that has experienced a decline in functioning is suddenly presented on stage and seen by others. The acting reminded her of the experience of being an active woman in a way that simultaneously contained the young and the old aspects of the self.

#### Theme 4: A shared expression

Almost all participants emphasized that playing in the life story of others required attunement to others in a way that led to a deep connection between the actor-participant and the teller. Rona (an 81-year-old woman) described the experience of playing in the story of Neomi, a young widow who raised three children alone:

“I felt a sense of sharing, just that one word—sharing…I mean, I entered this image, her being with her children and what she went through…, and how she was walking with them [in the street]. I just felt her… I really felt her.”

The deep encounter between the actor and the teller strengthened their mutual esteem and closeness. Reuven (a 80-year-old man) played Victor, a blind person, who traveled the world alone:

“So, I said—if he has that courage, I have to act it, to be like him, to show [what] he went through… I told him—well done! If you couldn’t see and went through all this… I appreciate him, and I love him…”

The ability to improvise and to perceive the other with empathic attunement enhanced the participants’ sense of connectedness and engagement with the group.

#### Theme 5: Challenges in the process

The theatrical expression in playback theatre seeks to reveal meaning thorough the aesthetic distillation of the experience in the theatrical improvisation. However, the interviewees explained that sometimes the theatrical improvisation was not sufficiently aesthetic, or did not exactly fit the teller’s subjective experience, as Benny (an 82-year-old man) explained:

“They were not professional actors…They performed fine, but sometimes it lacked a little… Professionals do it differently, with a lot of colors in it… But they tried…and each one did his part very nicely… I felt comfortable…that everyone knew each other, were good to each other and acted for each other.”

This quote demonstrates that even when the dramatic improvisation was not perceived as “artistic” enough, the participants could feel the actors’ sincere intention to be attuned to the story. The empathic attunement and the shared experience strengthened the sense of connectedness between participants and enhanced their social engagement in their own community.

### Expansion of social engagement: “We all joined together as a family, as friends”

This category describes the evolution of social engagement along the process and it reveals three themes: 1) A fusion of experiences, 2) Engagement with the ADC community, and 3) Engagement with the family. Additional supportive data for this category are presented in Table 5.

**Table 5:** Supportive data for category 3: Expansion of social engagement.

Theme	Quotes
**Theme 1: A fusion of experiences**	*It hurt when I heard it*. *I was really shaking*. *It made my body shake when she told all these things*.* *.* *.*I thought to myself*.* *.* *. *I had such a mother and she had such a father*. *I was really excited*, *very excited about what she was saying*.
	(Ilana, an 82-year-old woman)
	*It happened that I saw things [in the group] which happened to someone else*, *but it was similar to what happened to me in my life*. *I learned from that too*. *To get out of it and to move on*, *maybe in a better position*.* *.* *. *[In the improvisations] I acted parts of myself too*.* *.* *. *I played situations [for others] that I went through in my life too*.* *.* *.
	(Sara, an 82-year-old woman)
	Hedva, a 65-year-old woman watches the video where she plays in Rona’s story improvisation:
	*It's like my character*.* *.* *. *Miri (the teller) was like me too*. *She also was ashamed*, *and she also did not go with the boys*.* *.* *.*This was me (laughs)*, *I played myself*.* *.* *.
	*She went through things during the Holocaust*, *and all that*, *and here she talked about the fear and all that*, *what she is*.* *.* *. *and thousands of differences between her and me*, *but she*.* *.* *. *She also went through things*, *and what I have been through is similar*.* *.* *. (Josef, a 71-year-old man)
**Theme 2: Engagement with the adult day center community**	*Really*, *I know the people who were in the group better*, *even interpersonally*. *I see that there is more connection than there was before*. *After all*, *you can't know about a person [by] his appearance*. *What he was and what he thinks and what his marital status is*, *and how his worldview is*. *All this was revealed*. (Alina, a 74-year-old woman)
	*I would wait*. *Do you believe me*? *I was really waiting for the group*.* *.* *. *and I said—it's good*, *there's something to talk about*, *there's someone to listen to*.* *.* *.* *. *Yeah*, *I felt like I wasn't alone*. *At home*, *I felt we were done already*, *life was over*, *and I was alone*, *as if I was waiting*, *really waiting to follow him (the deceased husband)*. *I had no longer hope to continue living*, *and here I saw that there was a world*, *some people*, *I began to see things inside my heart*.* *.* *. *and I felt more confident*. *Slowly I began to get stronger*. *I said—no*, *it's not like I thought*, *it is not the end of the world*, *the end of life*. *There is more life to live*. (Rebeca, an 82-year-old woman)
	*What was amazing was that I think they discovered each other*, *found things they didn't know*. *Kind of created a fraternity; they became compassionate and supportive of each other*. *Even people you wouldn't believe would say such things to one another said very emotional things that helped a lot*.* *.* *. (Ruth, the social worker)
	*There is something about this group that worked once a week that is different from any other activity*, *we make*.* *.* *. *In the group connection*, *first of all*, *in their connection with each other*, *in modeling*. *Even in the model they got here*, *that they can share things—not only with the social worker*, *not only with the caregiver they love*, *but also with each other*. *Because there are people here who come every day*, *going through the daily routine in every course we offer*, *but they do not talk to each other*.* *.* *. *and we haven't had that before*. *This experience that they can suddenly realize they have here someone to be with*.* *.* *. *You can see that now couples sit together*. *Suddenly there is the opportunity to talk*, *to come in the morning and talk and not just have breakfast and continue with classes*. (Gital the social worker)
**Theme 3: Engagement with the family**	*They played it like what it was*. *Like what it was*, *what I was telling*, *exactly*. *I even said at home*, *they [my children] said*: *"What a beautiful thing you did*, *mother*.*" My kids*. (Tanya, an 84-year-old woman)
	Hava, a 78-year-old woman, at the end of the group process decided to write her life-story and to let her daughter read it for the first time in her life:
	.. *I let her [my daughter] read it*.* *.* *. *yes*, *I wanted to*. *I kept telling her*, *sorry*, *Lili*, *you have to forgive mother*.* *.* *. *She really felt it was authentic*, *and she told me*: *"Really*, *mother*? *That's how it happened and was it like that*?*"*
	*It's such a situation that you also see it from afar*,.* *.* *.*I was smiling*, *it was nice*, *it was beautiful*. *I told it to my sister and the kids*.* *.* *. *Yeah*, *and I showed them the picture [of the group]*, *and they were so happy*. *They said—you see*, *you always had to do things like this*. (Tina, a 76-year-old woman)
	*That's my feeling; it has accompanied me all my life*. *So now*, *at this age*, *only at this age I*.* *.* *. *when there are children and there are grandchildren*, *then slowly I open*.* *.* *. *I told my daughter*, *the older one*, *she lives in San Francisco*.* *.* *. *and my son*, *he lives in the kibbutz*.* *.* *. *I told them*. (Leora, an 84-year-old woman)

#### Theme 1: A fusion of experiences

During the co-creative process, most of the participants could identify their own personal experiences in the teller’s story in a manner that fused the theatrical improvisation with their own life stories to create a unified collective experience. Zohara (a 77-year-old woman) explained that the acting in Dorine’s story reminded of and connected her to her own beloved mother. While watching the video of herself playing the character of Dorine, she said:

Leora: “She [Dorine] is so good…she reminds me of my mother… she was also very good… she used to help people…who suffered from difficult situations in Iraq… She [my mother] used to helped them as much as she could…”

Interviewer: “So here, when you watch yourself in the video playing Dorine, you’re…”

Leora: “Like my mother, yes… here… she [I] was like my mother…”

This quote demonstrates how acting in the personal stories of others connected Leora to her own inner experiences and how the participants brought their own knowledge about the world into the improvisation. In this way, the theatrical improvisation created in the group is a fusion of the group participants’ life stories and experiences. This enhanced their sense of connectedness and engagement with the group, which was also reflected in the ADC community.

#### Theme 2: Engagement with the adult day center community

The participants indicated that the process created strong and caring connections in the group and, moreover, in the ADC community. Tina (a 76-year-old woman) shared the life-crossroads of the sudden death of her mother when she was seven years-old and her life-long longing for her mother. In the improvisation, the participants-actors wrapped the actor playing Tina in material as a metaphor for the care and love she wanted so much for herself. As a spectator of her own life-story, Tina said:

“I wish I had experienced the care they gave me here… I did not always have…another to embrace me [with love]… It was missing… But here in the group, they gave me a lot… we all joined together as a family, as friends.”

Through the improvisation, a new experience of “being embraced” with love and care was created. The participants indicated that the close and caring atmosphere was further reflected in the ADC’s routine outside the group’s process.

“What friends, what girlfriends… What fun, what fun, so nice, I love them so much and before the group I didn’t know them, I really didn’t know them. Each one in her corner, and that's it. . . . [and today] I enjoy coming to the club. ‘How are you? How do you feel? You didn’t come for two days.’ [There was a time when] no one would have said anything to me if I didn’t come for two days… and suddenly everyone noticed and felt I was missing.” (Marina, a 66-years-old woman)

Ruth, the ADC social worker, explains how the ability to embrace the other’s story through acting awakened positive attunement and strengthened the participants’ relationships in other activities in the ADC routine:

“Usually… everyone is sure he has the biggest problems in life, and that his aging process is the most difficult…Here in the group… you see that they are attuned to one another; they know how to empathize with each other, to feel compassion, to encourage, to give others time to tell their story and they even… perform for one another… “

Rosa, the housemother, continued:

“We could see it later when they sat together drinking the tea… you could see the connection that has been created… they used to say to each other ‘we should hurry drinking the tea, so we don’t miss the group.’”

As reflected in this quote, social engagement and the commitment to the group created a positive experience in the ADC community. It also encouraged the participants’ involvement in their own family.

#### Theme 3: Engagement with the family

The participants described how the process encouraged them to share their stories with their family and friends.

“There are many things I haven’t told. I didn’t want to hurt them…if they knew what I went through… with my stepmother during the difficult years… But now I started telling them, I told them I was playing in a group… ‘Mama, why didn’t you tell us all these years?’–‘What can I tell you? I didn’t want you to know or to hurt you.”(Ilana, an 82-years-old woman)

Being able to share their stories and express themselves promoted the older adults’ engagement in their own community as well as their family.

## Discussion

This study sought to develop an evidence-informed framework for playback theatre groups in ADC communities, as an intervention that integrates life-review with an improvised theatrical creative process. The integrative intervention presented here is founded on the solid evidence-based principles of the life-review in late life that is suitable for the aging population [60, 61]. The creative process focused on the exploration of life-crossroads stories [14], a short and original technique for conducting the life-review in a group setting. The uniqueness of this research also lies in the improvisational theatrical characteristic of playback theatre, which is based on a completely spontaneous co-creative playfulness and the engagement of others with the personal narrative [31, 83, 84]. Whereas the literature on creative arts interventions is lacking with aging-related evidence-informed models [13]; the findings of this study allow the transformative potential of this creative intervention to be captured. The findings portray three categories, which represent three types of potential transformative routes that may occur over the course of the process. Each route identifies and characterizes one aspect of the process’ mechanism and the way it might affect the participants. In this way, an evidence-informed framework for interventions [85] was developed, which is based on these three transformative routes.

Here, these potential transformations are described in terms of roles and role-repertoire. In drama therapy and psychodrama, the self is defined by the roles that the individual plays and expresses in daily life, as well as on the group stage, e.g.,–“a daughter,” “a father,” or “a therapist” [86, 87]. In the present context, the role “a child” or “an old woman” are parts of the human role repertoire. From this point of view, mental health is characterized by an ability to express a variety of roles, and the goal of the process is to enlarge the individual’s role-repertoire [86–88]. Accordingly, each transformative route refers to the potential of the process to expand the roles that were enacted and expressed in the group and in the community:

1. **The evolution of the life story:** This route points to the potential of the life story to evolve by means of the co-creative expression, which involves everyone in the group. This process adds the values of acknowledgment and testimony [89, 90], allowing new meanings and perspectives to co-evolve in an empathic, encouraging, creative and explorative manner. The results indicate that the process also helps resolve “unfinished business” from the past, an essential developmental task in old age [61, 91]. In this way, it had the potential to enhance the individual’s sense of integrity and acceptance of life [60, 61, 63].
  The life-crossroads stories, as self-defining memories, capture roles from the past that are essential for self-definition and meaning [14, 92]. The results suggest that reliving these roles on the group stage may help to preserve the individual’s sense of continuity and identity. At the same time, acting spontaneously on the group stage enables the participants to explore and experience new roles in the life story. For example, Simon’s life-crossroads story presented the traumatic loss of his brother and, by means of its performance, the role of “the guilty one” was transformed into the role of “a child” who wanted to help but could do nothing to change the terrible situation. In this sense, a new meaning, and a new role, were constructed in the group, and a more positive and adaptive story was created. Such a process may reshape the inner experience and induce psychological transformation [93–95].
2. **Evolution of playfulness:** This route points to the potential of the improvised play to connect the participants to their inner spontaneous-creative resources and thus to explore their potential for self-expression, meaning-making, and relationship-building [31, 32, 84, 96]. The creative process allows people to express the healthy and creative roles of active partners, such as “directors,” “actors,” and “playwrights,” rather than “illness- and dependence-related” roles. These new roles stimulate a sense of competence, purposefulness, and productivity that seems to awaken aspects of the individual’s past self from a time during which these aspects were more dominant. The experience of contributing to one’s own community is an essential aspect of mental health in late life [5, 97]. Such an experience has a precious value for the ADC members, most of whom have lost many of their social roles because of functional limitations and often feel irrelevant in society [97, 98]. This experience of competence may also generate a revival of the aging body [53], and the development of empathic attunement to others [46, 99]. This route points to the potential of the improvised acting to allow altered perceptions of the self and others.
3. **Expansion of social engagement:** This route points to the potential of the process to allow the participants to experience themselves as relevant and important to others and to relate to community roles such as a “friend,” “neighbor,” or “partner.” The ability to improvise and perceive the other with empathic attunement seems to enhance the participants’ sense of connectedness and engagement within the group. For people who facing the loneliness and social losses that are common in late life and particularly in the ADC community [100, 101], the expansion of such social roles in the group contributes to promoting a positive view of the self and others [102, 103]. Indeed, the intimate and shared atmosphere seems to have expanded to include the larger surrounding environment in the ADC community and in the participants’ families. Social involvement and close relationships in the community have a central role in preserving mental health in late life [104–106].

By combining the three transformative routes, a multi-dimensional perspective on the creative therapeutic process is created. Playback theatre groups seem to provide feasible opportunities for the participants to enlarge their role-repertoire and act upon multiple roles. This may, on the one hand, maintain a sense of continuity regarding past roles that are now being experienced again, and on the other hand, enable the individual to explore and experience new roles on the group stage, and also in life, fostering personal growth in the present. This dual contribution, the conservation of a sense of continuing and the ability to foster transformation and personal growth, is considered to be an anchor for preserving a positive identity in old age [107–109]. Furthermore, inspired by the ideas of Paulo Freire (1970) and Agusto Boal’s (1979), these transformative routs demonstrate how playback theatre groups, which is based on the participants’ life stories, reflective thoughts, creativity, images, and aging bodies—induced an experience of visibility, centrality, and purpose. These groups framework encouraged older adults to express their voices within the group and later within the ADC community and their families [110, 111].

The contribution of this research first lies in the development of a new evidence-informed intervention that can be applied in ADC communities, which integrates playback theatre and life-review principles, using the concept of life-crossroads. To the best of our knowledge, this is one of the first studies of playback theatre as a group intervention for older adults. While the literature on creative arts-based interventions in the community indicates its strong potential to promote the well-being of older adults [18, 19, 24, 26], methodologically sound research, which can bridge the gap between the healthcare sectors and the field of community-based arts and art therapies, is scarce [13]. The current study addresses this gap in that an evidence-informed framework was developed that allows older adults to bring up and explore their life-stories in a dramatic creative process in their own community, and that the transformative potential of the process was identified and characterized. The study results indicate the potential of this new integrative framework to serve as a creative intervention in ADC communities, as well as its potential to bring about a personal transformation and expand it to enable a person’s social engagement in the community.

### Limitations

The positive attention that was given to the participants during the intervention and the ensuing interview may have influenced their assessment of the intervention and may have biased the findings. To handle this influence and to maintain the data validity, various resources were used, and a group of researchers conducted an independent analysis of the data. In addition, in the last stage of the thematic analysis the data were presented to the participants and staff member for additional evaluation. Further, since the intervention includes life-review principles, which for people with memory problems can be difficult and distressing [112], we decided not to include participants with cognitive impairment. However, the new integrative intervention, which was found to have significant potential for promoting the engagement of the participants in the community, should include all visitors at ADCs in order to engage the community further [21, 113, 114]. Finally, the short-term nature of the intervention and the evaluation of its benefits immediately after termination, are also a concern. As mentioned, older adults who visit the ADC need consistent long-term support for wellbeing and quality of life, and its evaluation in the long term needs to be examined.

### Future research and clinical implications

This type of intervention is characterized by spontaneous, improvised actions, which may be both sensitive and intense [84, 115]. In addition, it involves the exploration of self-defining memories [92]. Therefore, one implication is that such interventions should be conducted by professional creative art therapists who are familiar with playback theatre and theatrical creative processes, as well as possessing an understanding of the life-review therapy and the psychological development process in old age [56, 57, 59].

The findings suggest diverse options for future research. First, future research would benefit from exploring the effect of the intervention with various aging populations, such as older adults with dementia or other neurodegenerative conditions, and in different care settings and institutions. Second, as playback theatre interventions have shown potential to affect positively the engagement of the participants in the community and group cohesiveness, such interventions could be further explored as a community-based intervention that includes ADC members, with staff and family members serving as participants [21]. Third, it is important to examine the experience of participating in a long-term intervention as a creative process that encourages learning and growth over time.

Fourth, thus far, only a few papers have related to the healing potential of playfulness in old age [31, 116]. Our findings, together with those of other preliminary studies [116, 117], indicate the need for future research to explore such interventions that involve playfulness and improvised acting in old age [31, 32]. Fifth, future research should inquire into the experience of the aging body in the realm of the creative theatrical process. The image of the aging body, as well as personal and social attitudes toward aging and cultural influences [118–120], might be positively influenced through playback theatre groups, which bring the aging body to the stage in a way that evokes and reinforces it [53], as our present findings suggest. Finally, this study explores the experience of a method that combines life-review with theatre, and a comparison of its value to that of other art modalities remains a worthy direction for future research.

## Conclusion

The objective of this study was to provide an evidence-informed framework for drama therapy interventions that can be applied in ADC communities, which integrates playback theatre and life-review principles. In addition, in the study we endeavored to understand the manner in which the process affects the participants. The findings portray three categories, which represent three types of potential transformative routes that facilitated social and creative engagement. The process was found to have the potential for two contributions, which are considered to be anchors for preserving older adults’ positive identity: maintaining a sense of continuity and fostering personal growth. This creative process can thus be conceptualized as an evidence-informed framework for creative interventions in ADC communities that integrates life-review and theatrical participation. In addition, this framework can be further be explored with additional aging populations and in different settings.

## Supporting information

S1 Table Description of the main stages of the intervention. (PDF)

S2 Table Interview guide with the groups' participants. (PDF)

S3 Table Interview guide for the focus groups with staff members. (PDF)
