# Supplementary material for: Playback theatre in adult day centers: A creative group intervention for community-dwelling older adults
Source: PLoS One. 2020 Oct 1;15(10):e0239812. doi: 10.1371/journal.pone.0239812 (PMC7529427; doi:10.1371/journal.pone.0239812)
Supplement: S1 Table — (PDF) [file pone.0239812.s001.pdf]

**S1 Table. Description of the main stages of the intervention**

| Stage | Main Goals | Method and tools |
|---|---|---|
| Stage 1: Introductory meetings with the group participants and with playback theatre (Sessions 1–2) | 1) Acquaintance among participants<br>2) Introduction to the ritual of playback theatre<br>3) Implementing the idea that the theatrical creative process is a safe space for bringing up and exploring personal experiences | • The process and the setting are explained to the group.<br>• A warm-up session is conducted to encourage the participants' playfulness and spontaneity.<br>• An introductory game is played to facilitate acquaintance among participants and to "awaken" their personal stories.<br>• The group is invited to create a theatrical improvisation that reflects one of the stories that emerged during the introductory game. One of the group participants becomes "the teller" and is invited to share a personal story with the group. The conductor can briefly interview the teller. The other group participants are instructed to use active listening skills—to remain open-minded and attentive to the personal story, as well as to any feelings and images that resonate within them.<br>• After the story has been told, the other group members, as playing-participants, create a theatrical improvisation echoing back the story. In this part, we use short, ready-made theatrical forms inspired by the "Playback Theatre short forms," developed by Fox and Salas (Rowe, 2007). Each short form has a unique definition of the theatrical elements, their position on stage, and the integration between them, such that the actors are enabled to transform the personal stories into a theatre piece.<br>• The teller witnesses and observes the improvisation from the point of view of a "spectator." At the end of the performance, guided by the conductor, the teller responds to the theatrical improvisation. At this stage of the process, he/she can also re-direct the improvisation and the way it reconstructs his/her story.<br>• The session ends with a sharing circle. The participants are invited to respond to the story and the theatrical improvisation from a subjective point of view, sharing more stories that are connected to the teller's story and the creative process. |
| Stage 2: Life-crossroads stories (Session 3) | 1) Understanding the concept of life-crossroads<br>2) Each participant identifies three to five life-crossroads in his/her life story | • Mythological stories that contain major changes in the hero's life-path are told in order to explain the concept of life-crossroads.<br>• The participants are asked to identify and write about three to five life-crossroads from their life story. Each participant is asked to write a few sentences describing events, thoughts, feelings, and significant persons that relate to each life-crossroads. Written materials are collected in a personal file.<br>• Participants receive hexagon-shaped, paper cards, with which to represent their life-crossroads (Keisari & Palgi, |

| | | |
|---|---|---|
| | | 2017). The hexagon represents the connection between life crossroads and the ability to move, change, replace, or isolate a single life crossroads in order to change one's perspective. The shape of the hexagon emphasizes the flexibility of the life story and the way people choose to tell their stories.<br>• Working in pairs, participants are asked to write the name of each life-crossroads on the hexagon-shaped cards and to construct the physical connection and links among the life-crossroads by positioning the cards in a way that represents their life trajectory.<br>• The session ends with a sharing circle. Each participant shares his experience during the process of identifying and writing about his/her life-crossroads. |
| Stage 3: Life-review on stage (Sessions 4-10) | 1) Observing one's life path from new points of view<br>2) Enhancing creativity and spontaneity<br>3) Rehearsing the idea that connecting and integrating one's life-crossroads create a more integrative and unified life-story | • A warm-up session is conducted to encourage the participants' playfulness and spontaneity.<br>• One of the participants becomes "the teller," sharing his/her life-crossroads story or several life-crossroads stories, which become integrated into one life-story.<br>• The group participants, with the help of the conductor, create an improvisation that reflects the life-crossroads stories' content. In this way, the theatrical improvisation created by the group usually simultaneously represents different phases of the life course – dramatic roles from childhood along with dramatic roles from adulthood and old age. The theatrical improvisation also strives to combine dramatic roles that represent achievements, positive memories, and coping resources together with dramatic roles that represent disappointments and harsh life events.<br>• The teller witnesses and observes the improvisation from the point of view of a "spectator." At the end of the performance, guided by the conductor, the teller responds to the theatrical improvisation. At this stage of the process, the teller can also re-direct the improvisation and the way it reconstructs his/her story.<br>• The session ends with a sharing circle. The participants are invited to respond to the story and the theatrical improvisation from a subjective point of view, sharing their personal stories that are associatively connected to the teller's story and the creative process. |
| Stage 4: Looking ahead (Session 11) | Choosing a future life-crossroads to strengthen one's sense of purpose and meaning in life | • A warm-up session is conducted to encourage the participants' playfulness and spontaneity.<br>• Participants are asked to choose and write a future life-crossroads, for example, studying a new skill or visiting a new/old place/friend. The participants share their choice with the group and add it to their life story woven in their personal filing cabinet. |

| | | |
|---|---|---|
| | | • On stage, the group again explores (for the last time) one of the participant’s life stories, adding dramatic representations of the new future life-crossroads.<br>• The teller witnesses and observes the improvisation from the point of view of a “spectator.” At the end of the performance, guided by the conductor, the teller responds to the theatrical improvisation. At this stage of the process, the teller can also re-direct the improvisation and the way it reconstructs his/her story.<br>• The session ends with a sharing circle. Each participant shares his/her experience during the process. Other personal stories are raised in the circle in response to the process. |
| Stage 5: Farewell meeting (Session 12) | 1) Summing up the process<br>2) Examining the group participants’ achievements<br>3) Farewell meeting | • Participants are asked to sum up the process and discuss their major achievements with the group by using the dramatic space: delivering a monologue, dedicating a poem or song to the group that expresses their feelings and thoughts, or simply sharing significant moments and achievements. The therapist also summarizes his/her own perspective of the significant moments of the therapeutic process for the participants. |
