# Supplementary material for: Playback theatre in adult day centers: A creative group intervention for community-dwelling older adults
Source: PLoS One. 2020 Oct 1;15(10):e0239812. doi: 10.1371/journal.pone.0239812 (PMC7529427; doi:10.1371/journal.pone.0239812)
Supplement: S2 Table — (PDF) [file pone.0239812.s002.pdf]

S2 Table. Interview guide with the groups' participants

Your experience of participating in a Playback Theatre group in an adult day center	• Describe your participation experience in the Playback Theatre group at the adult day center.<br>• Why did you join the process?<br>• What did you achieve through the process?<br>• Describe meaningful experiences from the process.<br>• Describe sessions or moments in the process that you remember, in particular.<br>• Which things interfered with you in the process?<br>• Describe challenging moments that were difficult for you in the process.<br>• How did the process affect you?<br>• In which day center's activities do you usually participate?<br>• How did the process affect your experience in the adult day center?
Your life-story in the group process	• Describe the experience of bringing your life-story to the group. Which life-crossroads stories did you choose to bring to the group process? Tell us about why you chose these stories.<br>Now, we will look at the video of the improvisation created by the group in response to your life-story.<br>• Describe your experience upon observing/watching the improvisation. What thoughts, feelings, emotions did you have?<br>• What do you feel about the improvisation?<br>• How did you feel in the group when they presented your story as a theatrical improvisation?<br>• How do you think the theatrical improvisation influenced the way you perceive your life-story?<br>• What was meaningful to you in the process of bringing the story to life on the group stage?<br>• Did anything about the group improvisation bother you? Which dramatic actions or choices did not accurately reflect your story?<br>• At the end of each session, the other participants responded to the story and shared the ways in which the story accurately reflected their personal experiences with the group. Tell us about this process for you. How did you feel during the sharing circle? How did others' sharing affect you and the way you perceive your life story?

Being a participant-actor	• Describe your experience of playing in the group process.<br>• Describe a meeting session in which you participated in an improvisation that was created in response to the life-story of another group participant. Describe your experience during this session.<br>Now, let's look at the videos that show you acting in a theatrical improvisation created in response to another participant's story:<br>• Describe your experience of acting in this improvisation. What thoughts and feelings do you have regarding the improvisation that was created by you and the others?<br>• Which qualities and skills were required from you as an actor on stage improvising in response to a personal story?<br>• Describe the dramatic actions and materials you used to respond to the story.<br>• Which challenges did you face during the improvisational creative process?<br>• What encouraged your participating in the theatrical improvisation?<br>• Describe the experience of being an actor on the group-stage. How did the dramatic acting affect you?
Group relationships	• Describe the relationships among the group participants.<br>• How did the process influence the relations among the participants?<br>• Describe the positive experiences in the process that relate to the group participants' relationships.<br>• Describe the events that took place over the course of the process which you had difficulty with. This may relate to having to deal with a conflict in the group participants' relationships.<br>• How did the presence of others in the group contribute to your personal process? How did their presence affect the way you told your story?<br>• Which parts of the process were meaningful to you in terms of your relationships with others? What made them meaningful to you?<br>• Consider the stories that were brought to the group by the other participants. How did these stories affect your experience?<br>• How did the process influence your experience as a member of the adult day center? In terms of your relationships with other members and staff members?
To summarize	• What other aspects of the process would you like to relate to?<br>• What is your opinion about continuing participating in such a group? In what way? Would you recommend that others take part in a similar process?
