# Supplementary material for: Playback theatre in adult day centers: A creative group intervention for community-dwelling older adults
Source: PLoS One. 2020 Oct 1;15(10):e0239812. doi: 10.1371/journal.pone.0239812 (PMC7529427; doi:10.1371/journal.pone.0239812)
Supplement: S3 Table — (PDF) [file pone.0239812.s003.pdf]

S3 Table. Interview guide for the focus groups with staff members

Playback Theatre group in the adult day center's daily routine	• For the last 12 weeks, a Playback Theatre group was facilitated at the adult day center. Describe the members' participation experience in the group from your own point of view.<br>• How did you experience the members' willingness to take part in the process?<br>• How was group perceived by the other members of the adult day center - the members who did not participate in the group?<br>• What can you tell me about what happened during the process?<br>• What did the members themselves tell you about the group?<br>• How was the group's presence reflected in the adult day center's daily routine?
The effect of the process on group participants – (refers to members who took part in the group)	• What is the extent of the participant's involvement in the adult day center's various activities? How did the group process affect their involvement?<br>• How much encouragement was required to get the participant to join the group? To continue participating in the group?<br>• How did the group process affect the participant?<br>• How did participation in the group influence his/her relationships with the other group members?<br>• What did the participant tell you about the process? How did he/she perceive it?
To summarize	• What other aspects of the process would you like to relate to?<br>• What format (process duration, duration/frequency of meetings, conductors, participants, setting) would you recommend for such a group process in adult day centers?
